# Long COVID in Children and Adolescents: Mechanisms, Symptoms, and Long-Term Impact on Health—A Comprehensive Review

**DOI:** 10.3390/jcm14020378

**Published:** 2025-01-09

**Authors:** Diana-Georgiana Basaca, Iulius Jugănaru, Oana Belei, Delia-Maria Nicoară, Raluca Asproniu, Emil Robert Stoicescu, Otilia Mărginean

**Affiliations:** 1 Disturbances of Growth and Development on Children Research Center, “Victor Babeș” University of Medicine and Pharmacy, 300041 Timișoara, Romania; diana.basaca@umft.ro (D.-G.B.); belei.oana@umft.ro (O.B.); marginean.otilia@umft.ro (O.M.); 2 Ph.D. School Department, ‘Victor Babeş’ University of Medicine and Pharmacy of Timisoara, 300041 Timisoara, Romania; raluca.asproniu@umft.ro; 3 Department XI Pediatrics, Discipline I Pediatrics, ‘Victor Babeş’ University of Medicine and Pharmacy of Timisoara, 300041 Timisoara, Romania; nicoara.delia@umft.ro; 4 Department of Pediatrics I, Children’s Emergency Hospital “Louis Turcanu”, 300011 Timisoara, Romania; 5 Research Center for Medical Communication, ‘Victor Babes’ University of Medicine and Pharmacy Timisoara, Eftimie Murgu Square No. 2, 300041 Timisoara, Romania; stoicescu.emil@umft.ro; 6 Department of Radiology and Medical Imaging, ‘Victor Babes’ University of Medicine and Pharmacy Timisoara, Eftimie Murgu Square No. 2, 300041 Timisoara, Romania

**Keywords:** long COVID, post-acute sequelae of SARS-CoV-2 infection, pediatric long COVID, children, adolescents, chronic COVID-19 symptoms, immune dysregulation, autonomic dysfunction, mitochondrial impairment, cognitive impairment, psychological impact

## Abstract

Long COVID, also known as post-acute sequelae of SARS-CoV-2 infection (PASC), is increasingly recognized as a condition affecting not only adults but also children and adolescents. While children often experience milder acute COVID-19 symptoms compared to adults, some develop persistent physical, psychological, and neurological symptoms lasting for weeks or months after initial infection. The most commonly reported symptoms include debilitating fatigue, respiratory issues, headaches, muscle pain, gastrointestinal disturbances, and cognitive difficulties, which significantly impact daily activities, schooling, and social interactions. Additionally, many children with long COVID experience psychological symptoms, such as anxiety, depression, mood swings, and irritability, likely exacerbated by prolonged illness and lifestyle disruptions. Risk factors for long COVID in children include pre-existing health conditions such as asthma, obesity, and neurological disorders, with adolescents and females seemingly more affected. Hypothesized mechanisms underlying long COVID include chronic immune dysregulation, persistent viral particles stimulating inflammation, autonomic nervous system dysfunction, and mitochondrial impairment, which may collectively contribute to the variety of observed symptoms. Long-term outcomes remain uncertain; however, long COVID can lead to school absenteeism, social withdrawal, and psychological distress, potentially affecting cognitive development. Severe cases may develop chronic conditions such as postural orthostatic tachycardia syndrome (POTS) and reduced exercise tolerance. This review synthesizes the existing literature on long COVID in children, examining its prevalence, symptomatology, risk factors, and potential mechanisms, with an emphasis on the need for further clinical studies. While existing research largely relies on surveys and self-reported data, clinical assessments are essential to accurately characterize long COVID in pediatric populations and to guide effective management strategies.

## 1. Introduction

Long COVID, or post-acute sequelae of SARS-CoV-2 infection (PASC), has emerged as a significant concern, not only in adults but also in children. The Center for Disease Control and Prevention define long COVID as illness extending beyond four weeks after the initial infection [1], while the World Health Organization describes it as ongoing or new symptoms appearing two months post-infection without another identifiable cause [2]. Although children are generally less severely affected by acute COVID-19, many develop persistent symptoms that can last weeks or months after the initial infection. Long COVID in children is characterized by a wide range of symptoms that significantly affect their physical, mental, and emotional well-being. This condition poses unique challenges for the accurate diagnosis, effective treatment, and the evaluation of long-term prognostic outcomes [3].

Children with long COVID may experience a wide range of symptoms that vary in severity and duration [4]. The most commonly reported symptoms include fatigue, which can be debilitating and impact daily activities, concentration, and school attendance [5]. Respiratory issues such as cough, shortness of breath, and chest pain are also frequently reported. Additionally, children may suffer from headaches, muscle and joint pain, and gastrointestinal disturbances, such as nausea, abdominal pain, and diarrhea. Neurological symptoms, including difficulty concentrating (often described as “brain fog”), dizziness, and memory problems, are particularly concerning because they can interfere with learning and cognitive development. Psychological symptoms such as anxiety, depression, mood swings, and irritability are also prevalent, likely exacerbated by the stress of prolonged illness and the disruption of normal childhood activities. Sleep disturbances, such as insomnia or excessive sleepiness, are common, further compounding fatigue and cognitive difficulties [3]. Some children also report the persistent loss of taste or smell, although these symptoms are less frequent in children than in adults [6].

While long COVID can affect children of any age and health status, certain factors may increase the risk of developing this condition. Children with pre-existing health conditions, such as asthma, obesity, or neurological disorders, may be more vulnerable to long COVID [7]. Adolescents appear to be more affected than younger children, possibly due to a combination of physiological and social factors. Additionally, the severity of the acute COVID-19 infection has been associated with a higher risk of developing long COVID, although children who had mild or asymptomatic infections can also experience prolonged symptoms [4]. Other potential risk factors include female gender and lower socioeconomic status, possibly due to disparities in healthcare access and support systems [4].

The exact mechanisms underlying long COVID in children remain unclear, although several theories have been proposed. Persistent viral particles or fragments may continue to stimulate the immune system, leading to ongoing inflammation that affects multiple organ systems. The dysregulation of the immune response, characterized by chronic inflammation and autoimmunity, could explain the wide variety of symptoms observed in children with long COVID. There is also evidence that long COVID may be related to autonomic dysfunction, particularly in children who experience dizziness, fatigue, and heart palpitations, suggesting that the virus may affect the nervous system in ways that are not yet fully understood. Another possible mechanism involves the effects of the virus on the vascular system, which may lead to microvascular damage and impaired blood flow to various organs, contributing to symptoms such as fatigue, headaches, and cognitive difficulties. Mitochondrial dysfunction, which affects the body’s energy production processes, has also been suggested as a possible contributor to the persistent fatigue and muscle pain seen in long COVID [8,9,10].

The long-term outcomes of long COVID in children are still being studied, but the condition has the potential to significantly impact a child’s quality of life [3]. Children with long COVID may struggle to return to normal activities, such as attending school, participating in sports, and socializing with peers. Prolonged physical symptoms, combined with psychological effects such as anxiety and depression, can hinder emotional and social development [11]. Moreover, educational setbacks due to concentration problems or missed school days may have lasting consequences on academic performance [4]. In more severe cases, children may develop chronic conditions, such as postural orthostatic tachycardia syndrome (POTS), which is often associated with fatigue, dizziness, and fainting [12,13]. Persistent respiratory symptoms can also lead to decreased exercise tolerance and reduced physical activity, which may contribute to deconditioning and further health problems [1,3]. One of the most concerning aspects of long COVID in children is the potential for ongoing and unpredictable health issues, as the condition may wax and wane over time, making recovery difficult to predict. While the conditions of many children with long COVID do improve over time, the duration of recovery varies, and some children may experience relapses or chronic health problems that extend into adolescence and adulthood [4,7].

The aim of this study is to investigate the long-term effects of SARS-CoV-2 infection in children and adolescents, focusing on both physical and psychological symptoms that persist beyond the acute phase of illness. Despite growing concerns about long COVID in children, there remain very few clinical studies that examine its full effects, with most focusing primarily on respiratory changes. Additionally, much of the current knowledge on lingering symptoms in pediatric cases comes from questionnaires or surveys rather than clinical assessments, highlighting a significant gap in objective, clinically verified data.

## 2. Materials and Methods

The article selection process for this literature review followed PRISMA (Preferred Reporting Items for Systematic Reviews and Meta-Analyses) guidelines [14]. In August 2024, we conducted a comprehensive search across PubMed, Google Scholar, Web of Science, and Scopus, for studies related to long COVID in children and adolescents, spanning publications from 2020 to 2024. The following keywords and Boolean logic were employed: “COVID-19” OR “SARS-CoV-2” OR “long-COVID” OR “post COVID” OR “post-acute” OR “persistent” AND “pediatric” OR “infant” OR “children” OR “adolescents”. Specialized terms were applied to specific sections as follows: “respiratory”, “cardiovascular”, “cardiac”, “gastrointestinal”, “psychology”, “mental health”, “neurology”, “motor”, “common motor syndrome”, “ENT”, “otorhinolaryngology”. Additional relevant studies were retrieved from references in key articles.

**Figure 1:**
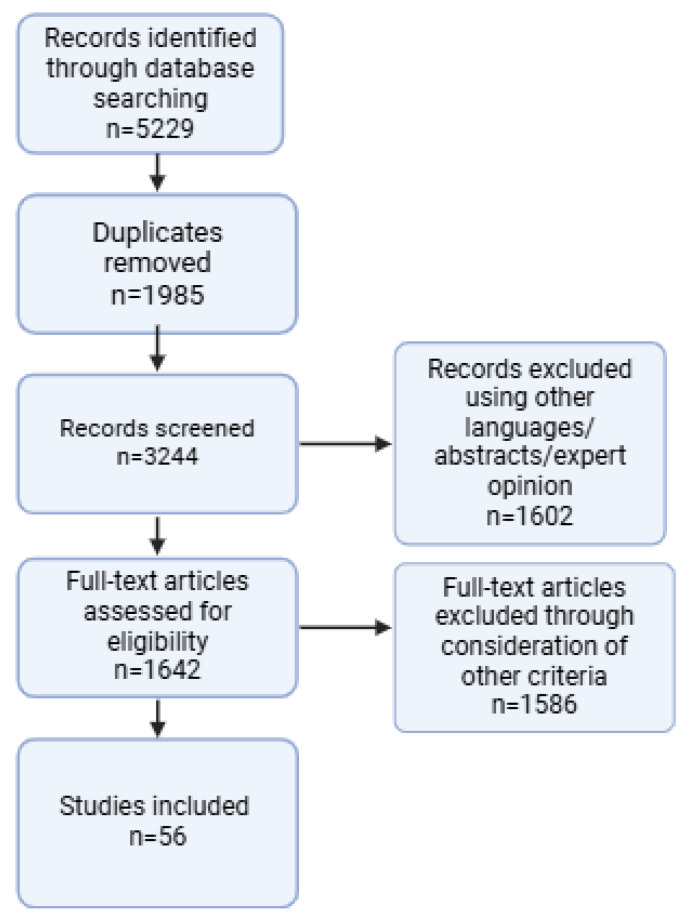
PRISMA flow diagram.

Articles were selected based on title and abstract, considering eligibility criteria including English language and a pediatric population (aged 0–18). Studies incorporated in the review included narrative and systematic reviews, longitudinal retrospective and prospective studies, and randomized controlled trials. Excluded studies included those in languages other than English, non-pediatric studies, expert opinions, and studies with adult populations. The final reference list emphasized originality and relevance to the scope of long COVID in children and its diverse symptoms. Figure 1 presents the search strategy and applied filters, structured according to the PRISMA statement.

## 3. Assessment of Long COVID in Children and Adolescents

**Figure 2:**
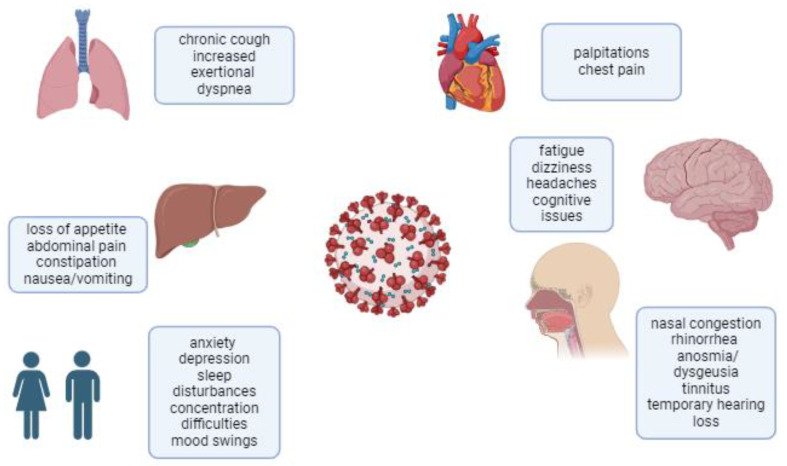
List of the most common symptoms of long COVID in children.

While children generally experience less severe COVID-19 symptoms than adults, emerging evidence indicates that some develop long COVID. This condition was first recognized in adults in early 2020, with pediatric cases increasingly documented over time [15]. The impact of long COVID on children can be substantial, leading to disruptions in schooling, social interactions, and physical activities. Understanding and managing long COVID in this population is challenging, given the wide spectrum of symptoms and the unique developmental considerations for children and adolescents [3]. Long COVID in children and adolescents involves a variety of mechanisms (Table 1), including immune dysregulation, autonomic dysfunction, and vascular abnormalities, leading to persistent symptoms across multiple systems (Figure 2).

The prevalence of long COVID in children varies by study, likely influenced by factors such as age, the severity of initial infection, and variants of the virus. Reports indicate that 1–45% of children with a history of COVID-19 may experience symptoms persisting for months [16,17,18]. Adolescents appear more susceptible than younger children, and those with pre-existing health conditions or severe initial infections are at higher risk. Large-scale studies from countries like the UK, the US, and Denmark have shown variable rates, often higher before the Omicron variant emerged, indicating potential differences in risk associated with different strains of SARS-CoV-2 [4,8].

The RECOVER-Pediatrics Group completed an observational research involving 5376 children ranging from 6 to 17 years old who were confirmed positive or negative with COVID-19. Subjects, divided into two age groups (6–11 and 12–17), identified 89 possible long-term COVID symptoms across 9 areas. The median time from COVID-19 diagnosis was 506 days for the first age group and 556 days for the second age group. Among positive participants, 45% of school-aged children and 39% of adolescents had more than one chronic symptom, as opposed to 33% and 27% in the uninfected groups, respectively. A PASC research index was developed based on the most common symptom clusters associated with disease, which were associated with poor health and quality of life. Clusters for younger children included pain, neurological, and gastrointestinal issues, whereas clusters for teens included odd taste or smell, pain, and exhaustion. Younger children had four symptom clusters: widespread symptom prevalence, headache with general discomfort, sleep disturbances with cognitive issues, and digestive symptoms. Adolescents were divided into three clusters: widespread symptom prevalence, diurnal sleepiness with pain, and poor smell or taste [16]. The researchers highlight that this was the first study to use a data-driven approach to explore differences in long COVID symptoms between school-age children (6–11 years) and adolescents (12–17 years), with slightly different symptoms among these groups [16].

### 3.1. Pulmonary Function and Symptoms Assessement

As COVID-19 frequently targets the respiratory system, persistent respiratory symptoms are relatively common post-infection. These symptoms may include chest pain, chronic cough, and increased exertional dyspnea, with some persisting for three months or more. For children aged six years and older experiencing prolonged symptoms, pulmonary function testing may be warranted to assess lung function and residual impact. The studies on long COVID in children indicated that 4–66% of children experienced post-acute COVID-19 symptoms, such as sleep disturbances, respiratory issues, nasal congestion, fatigue, muscle and joint pain, difficulty concentrating, and loss of smell and taste [19].

Adult long COVID syndrome commonly features abnormalities in pulmonary function tests or chest imaging in approximately 30% of cases [20]. In contrast, studies examining these function tests in children with long COVID are limited, and so far, findings have been minimal [21,22,23]. The initial study investigating the pulmonary manifestations of long COVID in children was conducted by Bottino et al. In this study, forced spirometry, the diffusing capacity of the lungs for carbon monoxide (DLCO), and lung ultrasound were assessed, highlighting that no pulmonary sequelae developed in children during follow-up [24]. A multicenter study indicated that COVID-19 has relatively mild, long-term respiratory effects in children, with a favorable outlook for full recovery [21]. Similarly, research by Dobkin et al. found that both spirometry and plethysmography results were normal in most pediatric long COVID cases, highlighting that significant respiratory impairment appears rare in this group [22].

In a study of 55 children with long COVID and 55 healthy controls, a restrictive lung pattern was found more frequently in the long COVID group. Children with long COVID showed significantly lower DLCO Z-scores, and predicted spirometry and lung volume values, though still within the normal range [25]. Spirometry and DLCO tests are valuable for evaluating functional capacity and identifying potential chronic lung effects in cases of cough, dyspnea, or chest pain.

Differences in study populations, confounding factors, and varying follow-up intervals contribute to the mixed results seen across studies, making it challenging to draw definitive conclusions on the respiratory impacts of long COVID in children. COVID-19’s pulmonary effects in children have also been evaluated through lung ultrasound (LUS), which offers a rapid, accessible, and non-invasive diagnostic option free of radiation exposure [26]. La Regina et al. used LUS to screen 607 children who had recovered from COVID-19, finding abnormal LUS results in only a small subset, further supporting the notion that severe or lasting lung impairment in children post-COVID-19 is uncommon [27].

Rinaldo et al. investigated 91 adult COVID-19 survivors using cardiopulmonary exercise testing, identifying an early anaerobic threshold as the most common abnormality, likely due to muscle deconditioning. In cases without lasting functional impairments, symptoms like fatigue and reduced exercise capacity may be attributed to muscle deconditioning and lifestyle changes caused by lockdowns and social isolation [28]. Jamaica Balderas et al. used radiological investigations and revealed interstitial and alveolar patterns in 27.9% and 21.4% of patients, respectively, with bilateral involvement in 23.7%. Follow-up evaluations showed improvement, but 13.5% still had interstitial patterns and 6.5% presented with pulmonary arterial hypertension, highlighting persistent respiratory and cardiac impacts. Symptoms were most prevalent in school-age children and adolescents [29].

Another study assessed fractional exhaled nitric oxide (FeNO) levels in children with long COVID, finding that FeNO values were lower in children with long-COVID syndrome than in those without symptoms, after adjusting for confounders. FeNO reflects nitric oxide from the proximal airways, influenced by inducible nitric oxide synthase (iNOS) activation by pro-inflammatory cytokines. Unlike alveolar nitric oxide (CaNO), which requires a multiple-flow technique, FeNO was measured with a single-breath technique suitable for children [30]. Lower FeNO levels could have supported the prolonged presence of the virus in the respiratory airways even months post-infection, possibly leading to minimal immune activation [30]. Nevertheless, the impact of nitric oxide production and levels during viral infections is still under investigation, and more extensive research is needed to clarify this relationship.

This study of a group of non-hospitalized COVID-19 patients reveals a lasting biomarker signature, with elevated levels of eotaxin, monocyte chemoattractant protein-1 (MCP-1), and interferon gamma-induced protein 10 (IP-10) persisting six months after mild infection compared to COVID-negative participants. However, these immune marker changes were not associated with persistent symptoms or respiratory disability. No specific immunological alterations correlated with post-COVID symptoms, and spirometry results were similar between COVID-positive and negative participants, regardless of symptom presence. This aligns with previous findings that respiratory complaints, like dyspnea, in post-COVID cases do not stem from impaired lung function or tissue repair issues [31]. Hypotheses for post-COVID conditions include antigen persistence, latent virus reactivation, chronic inflammation, and autoimmunity. In the study of Sommen et al. [31], elevated chemokines MCP-1, eotaxin, and IP-10 were noted, reflecting an immune response unique to COVID-19. While chemokines are crucial in the initial antiviral response, prolonged elevation may contribute to hyperinflammation and tissue damage, though no direct link to long-term respiratory symptoms was observed.

In an observational study of eight children with acute COVID-19, Denina et al. found that lung ultrasound results matched radiologic findings in the vast majority of participants, suggesting that LUS could be an effective method for detecting lung abnormalities in children with COVID-19 [32]. However, it is under-researched in the context of long COVID in children, even though it holds potential for detecting and monitoring subtle respiratory changes in this population. In a study of 104 pediatric COVID-19 patients, respiratory evaluations revealed minimal long-term pulmonary impairment. Despite 46.1% of participants experiencing respiratory symptoms during acute infection, chest auscultation results were largely normal, with only 13 cases presenting pathological sounds like rattles, crackles, or wheezes. Spirometry assessments were similarly reassuring, showing normal function for nearly all participants, with only two children displaying a reduced forced expiratory volume/forced vital capacity (FEV1/FVC) ratio that improved post-bronchodilation. LUS results were predominantly normal (73.3%), and although 27% showed mild abnormalities (LUS scores of 1 or 2), there were no indications of significant consolidation or ‘white lung’—an encouraging sign of mild residual changes. Notably, these LUS abnormalities tended to resolve over time, with the majority of patients showing normalization within three months [33]. In contrast, in the study of Grager et al., LUS findings were similar in 30 children with long COVID-19 and 15 children without any respiratory disorder. Additionally, there was not a substantial connection between the LUS findings and clinical data [34].

COVID-19-related LUS abnormalities may take time to resolve, even as respiratory symptoms and lung function improve. Continued LUS monitoring over subsequent months can help track recovery non-invasively, reserving X-rays and computer tomography (CT) scans for later or when essential. Observing LUS resolution patterns can shed light on the disease’s natural course and recovery mechanisms in children, potentially informing better post-COVID lung care and management strategies [35].

While most children exhibit normal lung function, subtle abnormalities in airflow and lung volume can persist, especially among those with respiratory symptoms. Techniques such as spirometry, DLCO, and LUS offer valuable insights into potential restrictive patterns or mild respiratory impairments that may not be immediately apparent.

### 3.2. Otorhinolaryngological Assesement

Otorhinolaryngologic issues are common in children with long COVID, including persistent nasal congestion, anosmia, and dysgeusia, which can significantly affect daily activities and quality of life. Anosmia and dysgeusia may last beyond the acute phase, with some children affected for over six months. Ear-related symptoms, such as ear fullness, tinnitus, and temporary hearing loss, are less frequent [36,37].

Hijazi et al. reported that, among 660 pediatric COVID-19 patients, the only otologic symptoms observed were ear pain and fullness, affecting 1.8% and 0.5% of cases, respectively [38]. A recent systematic review and meta-analysis on COVID-19’s otorhinolaryngological symptoms in children also indicated that hearing loss and vertigo were rarely observed [36]. Tufatulin et al., in a study of 87 children, found no instances of hearing loss or central auditory processing issues post-SARS-CoV-2 infection [37]. In contrast, Swain et al. noted that, out of 192 children with COVID-19, 10.4% experienced tinnitus, 8.3% reported hearing loss, and 4.2% presented with both symptoms [39]. Another study reviewed the medical records of 524 children to compare the rates of recurrent acute otitis media (rAOM) and post-ventilation tube otorrhea (VTO) between those with a history of COVID-19 and those without. Findings showed higher rates of rAOM and VTO in children with prior COVID-19 infection, suggesting a potential association. However, it remains unclear whether COVID-19 directly increases the risk of whether other factors like daycare attendance influence these outcomes [40].

Olfactory disorders, such as anosmia, hyposmia, and parosmia, have been identified as common symptoms of chronic COVID-19, with viral particles detected in the olfactory bulb of affected patients [41]. The infection of non-neuronal cells in the nasal epithelium, along with microvascular damage in the olfactory bulb, may account for these symptoms [42]. The findings suggest a direct and potentially severe impact on the olfactory pathway, with long-term neurological effects still to be fully explored. Most children regain their sense of taste and smell within six months; however, a small percentage, shown in the study of Mariani et al. [43]—2.3% for anosmia and 1.1% for dysgeusia—may continue to experience these symptoms for over 18 months.

In a study of 236 children with long COVID, anosmia/dysgeusia was reported as a symptom in 12.3% of cases [44]. Buonsenso et al. [45] evaluated the effects of chronic anosmia in children with long COVID. They found that 1.7% of these children continued to experience smell dysfunction at an average follow-up of over three months post-infection, impacting their quality of life. Older age and the persistence of additional symptoms—such as altered taste, dyspnea at rest and on exertion, chest pain, palpitations, and joint pain—were notably more common in the group with chronic smell impairment. Another study, conducted in Turkey, found that, in 8.4% of cases, anosmia persisted beyond one month post-infection [46]. One retrospective cohort study evaluated 92 children and adolescents face-to-face using a specially designed post-COVID-19 symptom assessment protocol conducted 1–3 months after COVID-19. Among the participants, 51% reported at least one persistent symptom, with loss of smell being one of the most prevalent. The clinical evaluation revealed that the most affected age group was children aged 10–18 years [47].

Highlighting that the range of symptoms in children with long COVID is highly variable, Miller et al. found in 4678 children that this disease could impact multiple systems, with eye–nose–throat symptoms (22.5%) being the most frequent after fatigue (27.5%) [48].

In children with long COVID, symptoms like sore throat, dysphonia, and dysphagia have been documented, though they occur at relatively low rates—approximately 2%, below 2%, and under 1%, respectively, as indicated by recent studies and meta-analyses [3,44]. The causes of ear, nose, and throat (ENT)-related symptoms in long COVID are still poorly understood, and research into specific ENT presentations and risk factors associated with pediatric long COVID is in its early stages. Complicating matters further, these symptoms are not exclusive to long COVID, making it difficult to isolate their direct link to the condition. However, persistent symptoms affecting smell, hearing, or nasal function after COVID-19 warrant a detailed assessment by an ENT specialist. Recommended diagnostic methods include fibroscopic evaluation and smell tests tailored to children, such as the U-Sniff Sniffin’ Sticks, to better assess and address long-term ENT effects in this population [49].

### 3.3. Gastrointestinal Assessment

Gastrointestinal (GI) symptoms are common in adults with long COVID, including abdominal pain, nausea, diarrhea, and altered appetite, which may persist for up to a year due to inflammation, gut microbiota disruption, or viral damage [50]. These symptoms can lead to fatigue, poor nutrient absorption, weight loss, and reduced quality of life [50,51]. In children, GI symptoms are less frequent and less severe than in adults, with reports of abdominal pain (2.91%), constipation (2.05%), nausea/vomiting (1.53%), and loss of appetite (6.07%), though they may still impact daily activities and overlap with fatigue and mood changes [3].

An US-based study investigated a large cohort of patients under eighteen, which reveals a marked increase in GI risks associated with COVID-19. Findings show a higher likelihood of experiencing symptoms like abdominal pain, bloating, constipation, diarrhea, nausea, vomiting, and gastroesophageal reflux disease (GERD). COVID-19 was also associated with a greater risk of at least one GI symptom, disorder, or a related healthcare visit during both post-acute and chronic stages. Brodi et al. [10] suggest that, in some children, the virus may persist in the gut instead of being completely cleared, allowing it to continue damaging the intestinal lining and contributing to gastrointestinal issues. This viral persistence might also trigger an autoimmune response in the circulation, leading to additional symptoms.

A possible biological mechanism linking COVID-19 to gastrointestinal symptoms is the high concentration of angiotensin-converting enzyme 2 (ACE2) receptors on the border of the small intestine, which SARS-CoV-2 uses to enter host cells [52]. This interaction disrupts normal gut functions, potentially leading to inflammation and GI symptoms. Beyond direct infection, SARS-CoV-2 has been shown to alter the gut microbiome, with studies indicating that these microbiome disruptions may extend into the long-term post-acute phase [53,54]. Additionally, research has identified prolonged viral shedding in feces and the persistent presence of the virus within the GI tract, further supporting the relationship between COVID-19 infection and ongoing gastrointestinal effects [55]. These findings suggest that the virus may continue to impact gut health even after respiratory symptoms subside, contributing to the GI symptoms observed in long COVID. Alterations in the gut microbiota may also contribute to the persistence of respiratory and neurological symptoms. In support of this, Mendes-Almeida et al. [56] showed that transplanting gut bacteria from long COVID patients into healthy mice led to cognitive impairment and weakened lung defenses in the mice, effects that were partially alleviated by probiotic treatment.

The study of Cooper et al. [57] observed two distinct patterns of post-COVID-19 liver injury in pediatric patients: acute liver failure requiring transplantation in infants, and hepatitis with cholestasis in older children, with both types showing inflammation and bile duct proliferation on biopsy.

### 3.4. Cardiological Assessment

In adults with long COVID, cardiovascular symptoms such as chest pain, palpitations, shortness of breath, and exercise intolerance are frequently reported. Studies indicate these may result from lingering inflammation, endothelial dysfunction, or direct viral effects on the heart and vasculature. Cardiologic assessments often include echocardiography, cardiac MRI, and biomarkers such as troponin and N-terminal pro–B-type natriuretic peptide (NT-proBNP), which can reveal myocarditis, pericarditis, or subtle myocardial dysfunction. Some adults also exhibit POTS and other dysautonomia symptoms, highlighting the virus’s potential impact on autonomic regulation [58,59].

In children, the presentation of cardiovascular symptoms in long COVID appears less severe but still notable, particularly in cases with multisystem inflammatory syndrome in children (MIS-C) following acute infection. Pediatric patients may experience palpitations, chest pain, fatigue, and mild dyspnea, although these are typically milder and often resolve over time. Echocardiographic assessments in children have occasionally shown transient ventricular dysfunction or coronary artery changes, particularly in MIS-C cases. Unlike adults, severe outcomes are rarer in children, though ongoing monitoring is essential for detecting any lasting cardiac impact as they recover [58,59,60].

The study of Sabatino et al. [61] on 157 previously healthy children with prior asymptomatic or mild COVID-19 revealed subtle but notable reductions in left ventricular (LV) global longitudinal strain (GLS) compared to healthy controls, indicating mild, subclinical LV systolic impairment. While all GLS values remained within normal ranges, 7% of children had early signs of LV dysfunction. No correlation between immune response markers and GLS was found, suggesting these myocardial changes may result from chronic inflammation or autoimmune effects rather than initial immune strength. Another study underscores the importance of cardiopulmonary exercise testing (CPET) as a diagnostic tool to investigate the potential underlying causes for persistent symptoms in children with long COVID, even when standard evaluations for cardiopulmonary or upper airway disease are normal. Among the 23 children, the most commonly reported symptoms were dyspnea on exertion, chest pain, and dizziness. CPET revealed that nearly half of the participants showed decreased exercise capacity, primarily attributed to deconditioning, body habitus, and bronchospasm, with additional factors such as ventilation–perfusion mismatch and voluntary hyperventilation [59].

In the study of 56 children with long COVID compared to 27 healthy controls, researchers found no significant differences in left ventricular function but did observe slightly prolonged QTc intervals (within normal range) in the long COVID group. Heart rate variability (HRV) analysis indicated altered autonomic function, with increased parasympathetic activity and reduced r-MSSD values, suggesting autonomic regulatory abnormalities potentially linked to symptoms like palpitations, dyspnea, and fatigue [58]. These findings highlight autonomic dysfunction as a possible driver of cardiac-related symptoms in pediatric long COVID. The study of Erol et al. [62] investigated persistent symptoms in children following COVID-19, with a particular focus on evaluating cardiac symptoms through clinical assessments, including blood pressure measurements, electrocardiography, and echocardiography. The findings revealed statistically significant differences in systolic blood pressure, left ventricular ejection fraction, relative wall thickness, and tricuspid annular plane systolic excursion between children with prior COVID-19 and healthy controls. Ashkenazi-Hoffnung et al. evaluated 90 children, highlighting that persistent symptoms, especially in those over the age of 11, included fatigue (71.1%), dyspnea (50%), and myalgia (45.6%). Respiratory assessments revealed mild obstructive patterns in 45% of cases, with many showing reversibility after bronchodilators. Cardiac evaluations were mostly normal, though two adolescents exhibited transient electrocardiographic abnormalities and mild findings on cardiac MRI [63]. Of the 82 children included in another study, 21% demonstrated electrocardiographic changes, with 9% showing significant abnormalities, though none had corresponding echocardiographic findings, and most changes resolved over time [64].

A longitudinal study showed that elevated BNP and troponin levels predicted ventricular dysfunction at admission, peaking early (day 1 for troponin, day 3 for BNP) and typically normalizing within two weeks, though BNP rose transiently after intravenous immunoglobulin (IVIG), likely due to volume load. Approximately a third of patients showed left ventricular systolic dysfunction at admission, generally resolving by discharge or shortly afterward, though mild mitral regurgitation persisted longer, suggesting potential subclinical myocardial injury that self-resolves over several months. Diastolic dysfunction occurred in about 34% of cases and persisted in a smaller subset for up to six months, indicating a slower recovery compared to systolic function. Coronary abnormalities were mostly mild and transient, except one case with a giant aneurysm persisting to the one-year follow-up. PR interval prolongation and ST-T wave changes were common but typically resolved within two weeks, with only minimal long-term impact as four patients showed persistent PR prolongation at one year, improving with exercise. Exercise testing showed normal results for all patients, suggesting that MIS-C rarely leads to exercise intolerance. During follow up (4–6 months after discharge), 24 patients underwent CMR, with findings showing that 20.8% had an enlarged left atrial volume and 4.2% an abnormal LV end-diastolic volume index. The median left ventricular ejection fraction (LVEF) on cardiovascular magnetic resonance (CMR) was 57.5%, with 12.5% of patients showing reduced LVEF. The median right ventricular ejection fraction (RVEF) was 54%, with no patients displaying abnormal right ventricular function. Among the 29 patients who qualified for graded exercise tolerance test (GXT) at 4–6 months post-discharge, all showed normal exercise capacity and no ventricular ectopy, while 5% had an initial abnormal PR interval that normalized with exercise [60].

### 3.5. Metabolic Assessment

Metabolic changes in children with long COVID show patterns that suggest potential alterations in energy regulation, lipid metabolism, and inflammation. While detailed studies on this topic in children remain limited, emerging data highlight several metabolic disturbances.

One noted metabolic effect in children with long COVID is a tendency towards altered lipid profiles, including elevated levels of triglycerides and LDL cholesterol in some cases. This shift may indicate disruptions in lipid metabolism, potentially linked to prolonged low-grade inflammation or viral effects on lipid-regulating pathways. In the study of an adult cohort, they demonstrated elevated risks for several dyslipidemia outcomes compared to the control group. Elevated levels of total cholesterol, triglycerides, low-density lipoprotein (LDL), and decreased high-density lipoprotein (HDL) were observed, with each parameter showing a significant hazard ratio increase. Furthermore, COVID-19 patients showed an increased likelihood of a significant body-mass index (BMI) rise and lipid level thresholds across various demographic subgroups [65]. In a follow-up study of 1413 children who had recovered from COVID-19, 20% showed persistent symptoms, primarily including fatigue, difficulty with concentration, sleep disorders, headaches, and muscle pain, with a higher prevalence among older children and those with a higher BMI [66].

Additionally, insulin sensitivity appears affected in certain cases, with some children showing signs of insulin resistance. Barberis et al. [67] used metabolomic methods to uncover lipid biomarkers in COVID-19 patients, supporting the occurrence of insulin resistance after infection. Similarly, another study found that hyperglycemia often accompanies severe respiratory symptoms in COVID-19 cases, highlighting insulin resistance as a primary contributing factor [68]. These changes can contribute to symptoms such as fatigue, reduced energy levels, and altered weight gain or loss, complicating recovery and return to normal activity levels. From the start of the COVID-19 pandemic, evidence has shown that COVID-19 patients have higher rates of hyperglycemia, newly diagnosed diabetes, and increased need for pharmacologic treatment. Studies are actively exploring post-COVID changes that may lead to glucose intolerance, with three main mechanisms emerging: an increased proinflammatory state, the involvement of ACE-2 receptors, and pancreatic beta cell alterations [69].

A study following children and adolescents aged 10–19 years without prior diabetes or high HbA1c levels investigated the long-term links between COVID-19 and type 2 diabetes mellitus (T2DM) risk. Results showed a consistently higher risk of developing T2DM in children post-COVID-19 across follow-up periods of 1, 3, and 6 months. In children with overweight or obesity, COVID-19 was associated with even higher relative risks for T2DM. Analyses excluding early-onset T2D cases confirmed COVID-19’s association with increased T2D risk across different time frames [70]. Another study declared that no new-onset DM was observed among pediatric patients with confirmed COVID-19 or in the control group during both initial and subsequent follow-up visits, despite the comprehensive testing of fasting blood glucose and A1C levels [71].

In terms of inflammatory markers, long COVID children often have elevated cytokines and other markers of systemic inflammation, which may drive metabolic stress and affect glucose metabolism. This chronic inflammation may disrupt normal endocrine functions, impacting growth and development, as well as overall metabolic balance. Finally, mitochondrial function may be altered in long COVID, as suggested by fatigue and exercise intolerance commonly reported among affected children, signaling potential long-term impacts on cellular energy production. Systemic inflammation and cytokine activity associated with long COVID may play a role in appetite-related symptoms. Recent findings indicate that children previously infected with COVID-19 had a 14% increased likelihood of experiencing appetite issues compared to those without infection. This highlights how inflammatory responses, particularly cytokines, may disrupt appetite regulation, which resembles the appetite loss observed in depressive conditions [72,73]. Multisystem inflammatory syndrome in children (MIS-C) is a febrile inflammatory condition in children that can arise weeks following SARS-CoV-2 infection or exposure. It is characterized by widespread inflammation and affects multiple organs, leading to significant cardiovascular, gastrointestinal, and neurological symptoms. Children with MIS-C often experience intense abdominal pain, GI symptoms, myocardial dysfunction, neurological abnormalities, and kidney involvement [74].

### 3.6. Mental Health, Psychological, and Behavioral Assessment

Children experiencing long COVID are increasingly noted to have mental health challenges, including anxiety, depression, sleep disturbances, concentration difficulties, and mood swings. Research indicates that these symptoms may be attributed to several potential factors [11,75].

Firstly, systemic inflammation triggered by SARS-CoV-2 is believed to play a significant role. Elevated cytokine levels, a part of the body’s immune response, are associated with neuroinflammation, which may influence mood, cognition, and overall mental health. This inflammatory response can disturb neurotransmitter systems, particularly those involved in regulating mood and stress responses, leading to symptoms such as anxiety and depression [60,69].

Secondly, disruptions to the central nervous system (CNS) are thought to be a possible cause. Studies on adults with long COVID reveal that the virus may directly or indirectly impact the brain, which could similarly affect pediatric patients. The hypothalamic-pituitary–adrenal axis, a system involved in stress response, can be altered by viral infections, potentially leading to lasting impacts on mental health [76].

Psychosocial factors also contribute significantly. Isolation, reduced physical activity, and disruptions in routine due to quarantine or hospital stays can compound stress and increase vulnerability to mental health issues [75]. Additionally, children face social and educational disruptions, which may exacerbate feelings of anxiety and impact cognitive function, such as memory and focus [77].

Before, it was uncertain to what degree SARS-CoV-2 infection specifically contributed to worsening mental health in children, separate from general pandemic impacts. Among children with prior COVID-19 infection, the pooled prevalence of mental health issues ranged from 5% for appetite disturbances to 15% for depression. These figures are higher than pre-pandemic global estimates for children’s mental health issues (13.4% total, 6.5% for anxiety, 2.6% for depression) but the fall below the elevated levels reported for anxiety (20.5%) and depression (25.2%) during the pandemic [72,78].

A systematic review and meta-analysis highlighted increased mental health issues among children with prior COVID-19 infection, particularly anxiety and depression. Compared to non-infected controls, children with COVID-19 had over twice the odds of experiencing anxiety (9%) and depression (15%). Appetite problems also emerged, with a 5% prevalence and 14% higher odds in previously infected children, underscoring long COVID’s potential impact on eating behaviors. Other symptoms, such as concentration issues (6%), sleep disturbances (9%), and mood swings (13%), were observed but showed no significant difference from controls [79]. In the study of Buonsenso et al. [11], they noted a high prevalence of neuropsychiatric symptoms in children with persistent post-COVID issues. These included concentration difficulties (60.6%), memory issues (45.9%), trouble with daily activities (40%), information processing challenges (32.7%), and short-term memory problems (32.7%). More than half (54.7%) exhibited at least three mental health symptoms, 8.8% had two, 10.6% reported one, while 25.9% showed no symptoms. Of those without pre-existing conditions, only 28.7% reported no mental health or cognitive issues following COVID-19 [11].

### 3.7. Motor Disorders Assessment

In children with long COVID, common motor symptoms and syndromes often include generalized fatigue (10%), the most common, followed by muscle weakness, tremors, and difficulty with coordination [80]. These motor issues can interfere with daily activities, such as walking, writing, and even basic tasks, and may persist for weeks or months after the initial infection.

One of the primary causes of motor symptoms in long COVID is believed to be related to inflammation in muscles and nerves. The heightened immune response triggered by the infection can result in muscle pain and inflammation, sometimes leading to myalgia and, in severe cases, myositis. This inflammation can impair muscle function and contribute to fatigue and weakness, which are commonly reported in long COVID [81,82].

Neurological effects also play a significant role. Long COVID can impact the central and peripheral nervous systems, potentially disrupting communication between the brain and muscles. For instance, children may experience tremors or involuntary muscle contractions due to the misfiring of nerve signals [83,84]. In severe cases, these issues can resemble motor syndromes seen in other neurological conditions, such as tics or even dystonia, which involve involuntary, repetitive movements [85].

Vascular changes and reduced blood flow to muscles and nerves may contribute to symptoms as well, leading to issues with coordination and endurance [83,86]. Some children also report difficulties with balance and proprioception, likely due to these combined neurological and vascular effects [87].

Lastly, prolonged immobility and reduced physical activity during illness or quarantine can exacerbate muscle weakness and fatigue, compounding motor symptoms in recovery [77,88].

Common motor syndromes in children with long COVID remain relatively understudied and can present with considerable variation. These motor symptoms might range from mild coordination issues to more noticeable changes, such as tremors or muscle weakness. While some children may experience transient symptoms, others might see them persist longer, but limited research makes it challenging to draw definitive conclusions on prevalence and duration [3,80]. In a study, sore muscles were reported in 9% of COVID-positive participants compared to 3% in the COVID-negative group, while fatigue was present in 31% of the COVID-positive group compared to 13% of those who were negative [89]. In another analysis, the few reported motor symptoms, such as myalgia and arthralgia, show a prevalence of around 3.76%. These symptoms are notable as they may indicate broader systemic involvement, possibly resulting from inflammation or immune dysregulation after COVID-19 infection. However, the lack of detailed research and varied symptom presentation complicates a definitive understanding of the frequency and severity of motor issues in pediatric long COVID [3].

### 3.8. Neurological Assessment

Long COVID in children is associated with various neurological symptoms, including headaches (3–80%), dizziness (3–20%), cognitive issues (12–70%), and even more persistent conditions such as brain fog and memory problems (2–81%) [90]. Emerging evidence points to several mechanisms that may underlie these neurological effects in pediatric long COVID patients [76,91].

One potential factor is neuroinflammation. SARS-CoV-2 infection can trigger a robust immune response, leading to elevated levels of cytokines and other inflammatory markers in the brain. This inflammation may interfere with normal brain function, potentially causing cognitive symptoms like memory issues, concentration difficulties, and slower information processing [76].

Another mechanism is related to direct or indirect effects on the central nervous system (CNS). While SARS-CoV-2 is primarily a respiratory virus, studies suggest it can impact the brain either through direct viral invasion or indirectly via immune-mediated damage to nerve cells. The blood–brain barrier might also be compromised, allowing harmful substances or immune cells to affect brain tissue [76].

Vascular changes are also thought to play a role. COVID-19 has been shown to affect blood vessels and increase the risk of microvascular complications, potentially disrupting blood flow to the brain. In children, this may manifest as frequent headaches, dizziness, and fatigue due to reduced oxygen delivery to brain regions involved in alertness and focus [92]. Psychological stress and lifestyle disruptions further contribute to neurological symptoms. Extended periods of isolation, reduced physical activity, and anxiety surrounding illness can heighten stress, leading to mental fatigue and, potentially, the worsening of cognitive symptoms.

A study of 322 children and adolescents investigated long-term neurological symptoms. Initially, 60% displayed symptoms, which dropped to 20% at one month and then leveled off to 22% after 3–5 months. Persistent neurological symptoms included headaches, fatigue, and loss of smell. Among younger children (1.5–5 years), internalizing behaviors were noted, while in older children (6–18 years), anxiety and post-traumatic stress were the most prevalent [75]. Memory issues have been frequently noted in children with long COVID, with prevalence estimates ranging from 10.1% to 18% [76,93]. Recovery periods vary significantly, spanning from two weeks up to six months, and cognitive difficulties are more often observed in older children [17,94]. Although the exact causes are not fully understood, possible explanations include systemic inflammation, the disruption of the blood–brain barrier, or the activation of brain microglia.

Headaches are common in the general population and may not always be linked to COVID-19. However, symptoms like ageusia and anosmia, strongly associated with COVID-related headaches, provide important insights into the infection’s neurological effects [92]. One meta-analysis reported an overall prevalence of 35%, which decreased to 5% in controlled studies [95], while another estimated a pooled prevalence of 7.8% [3]. Buonsenso et al. [96] noted that symptoms persisted for an average of 8 months in children.

Fatigue is a consistently reported symptom with prevalence rates varying from 3% to 87% [76]. Older children, especially those aged 6–17, are more frequently affected [69]. The mechanisms behind fatigue are likely multifactorial, involving systemic inflammation, sleep disruption, and psychological stress [97,98]. Similar to Chronic Fatigue Syndrome, inflammation may impact areas of the brain, such as the hypothalamic paraventricular nucleus, potentially triggering an exaggerated stress response in vulnerable individuals [99]. Dizziness, although less common, affects around 3–20% of children with long COVID, with meta-analyses suggesting a prevalence of approximately 4% [3,17,90]. While often brief, dizziness can persist for up to 8 months in some cases and may involve neural dysfunction and immune responses [11].

Most of these studies followed up with patients for a maximum of six months. However, due to the short follow-up period and the disease’s recent emergence, understanding the full scope of COVID-19’s potential future neurological, cognitive, and neuropsychiatric effects remains challenging.

## 4. Limitations

A limitation of this study also lies in the reliance on subjective data collected through questionnaires and surveys. These self-reported assessments, while valuable for capturing a broad spectrum of symptoms, are inherently prone to biases, such as recall bias, the misinterpretation of questions, or the influence of personal and cultural perceptions. In the context of pediatric long COVID, these issues are particularly relevant, as children may have difficulty accurately articulating their symptoms, leading to potential underreporting or the overestimation of certain conditions. Additionally, the subjective nature of these tools limits the ability to objectively verify symptoms or establish clear causal relationships between reported symptoms and underlying mechanisms. Despite these limitations, questionnaires and surveys remain valuable for large-scale studies, allowing for the collection of data from diverse children and adolescent populations.

## 5. Conclusions

This narrative review highlights the wide-ranging impact of long COVID on children and adolescents, including persistent symptoms across respiratory, cardiovascular, neurological, gastrointestinal, and psychological systems. Children, though often less severely affected by acute COVID-19 than adults, can experience enduring symptoms that disrupt daily life and well-being. Potential mechanisms for these symptoms include immune dysregulation, autonomic dysfunction, and vascular changes, though more research is needed to clarify their roles. Despite growing attention to pediatric long COVID, much remains unknown about its long-term effects, highlighting the need for extended studies and standardized clinical assessments to guide effective interventions and support for affected children.

## Figures and Tables

**Table 1:** Mechanisms and associated symptoms of long COVID in children and adolescents.

Mechanism	Effects
Immune dysregulation	Autoimmunity and hyperinflammation,
Vascular dysfunction	Reduced blood flow and potential micro-clots
Autonomic nervous system dysfunction	Altered heart rate, blood pressure, and digestive responses
Neurological impacts	Direct effects on brain and nerves
Mitochondrial dysfunction	Reduced cellular energy production
Endocrine disruptions	Altered hormonal balance and metabolism
Pulmonary impacts	Persistent lung inflammation or fibrosis
GI microbiome alterations	Changes in gut bacteria affecting digestion and mood
Psychological stress	Anxiety and stress from infection and isolation

## Data Availability

No new data created.
